# Critical Evaluation of the Efficiency of Colorectal Fellowship Websites: Cross-sectional Study

**DOI:** 10.2196/30736

**Published:** 2021-10-15

**Authors:** Qi Yan, Katherine Jensen, Alyssa Field, Christian Goei, Haisar E Dao Campi, Alicia Logue, W Brian Perry, Mark G Davies

**Affiliations:** 1 Department of Surgery University of Texas Health Science Center at San Antonio San Antonio, TX United States; 2 University of Texas Health Science Center at Houston San Antonio, TX United States

**Keywords:** recruitment, GME, social media, websites, content, accessibility, online information, fellowship information, colorectal, graduate education, graduate medical education

## Abstract

**Background:**

Websites are an important source of information for fellowship applicants, as they can influence ongoing interest and potential program selection.

**Objective:**

This study aims to evaluate the current state of colorectal fellowship websites.

**Methods:**

This cross-sectional study evaluates the quantity and quality of information available on websites of colorectal fellowship programs verified by the Accreditation Council for Graduate Medical Education in 2019.

**Results:**

A total of 63 colorectal fellowships were included for evaluation. Websites were surveyed for content items that previous studies have found to be influential to program applicants. The 58 (91%) programs with a functional website were evaluated using an information index (calculated as a function of availability of content items concerning education, application, personnel, and benefits) and an interactive index (calculated as a function of accessibility and usability of the webpage). Programs had a median total score of 27.8 (IQR 21.5-34.5) of 79. The median score for the interactive index was 7.5 of 15 and for the information index was 20 of 64. The median scores for website application, education, personnel, and benefits or life considerations were 5, 5.5, 3.3, and 4 of 13, 24, 13, and 14, respectively. There was no difference in total score between programs in different geographical regions (*P*=.46).

**Conclusions:**

Currently, colorectal surgery fellowship program websites do not provide enough content for applicants to make informed decisions. All training programs, regardless of specialty, should evaluate and improve their digital footprint to ensure their websites are accessible and provide the information desired by applicants.

## Introduction

Residency and fellowship training program websites often serve as a first impression and vital source of information for applicants. It is a resource for training programs to attract highly competitive applicants and for applicants to identify best fit programs.

As early as 1998, program websites were found to influence nearly three-quarters of emergency medicine applicants [1,2]. In 2011, a study on anesthesia applicants found that 98% of applicants visited program websites during their residency application process. Program websites have been found to influence where applicants apply and decide to interview; how applicants prepare for interviews; and, ultimately, the formulation of rank lists [2-5]. Thus, a functional and informative website is a necessary promotional tool for graduate medical education (GME) programs to attract interested applicants. However, evaluation of selected specialties including vascular surgery, general surgery, orthopedic spine surgery, and radiology have found training websites to be lacking in quality, content, and design [6-11].

Colorectal surgery is a popular field. The number of applicants to colorectal surgery fellowship programs increases with each passing year. The application and recruitment process is time-consuming and costly for both programs and applicants. Well-maintained program websites can facilitate this process for both parties, enabling programs and applicants in finding their best match. Colorectal training websites, however, have not been previously evaluated.

The aim of this study is to evaluate the accessibility, content, and design of colorectal fellowship websites.

## Methods

A cross-sectional review of Accreditation Council for Graduate Medical Education (ACGME)–approved colorectal fellowship program websites was conducted using a validated assessment tool with two single-blinded reviewers [7].

### Setting and Participants

A list of colorectal fellowship training programs within the United States was obtained from the ACGME program list in September 2019 [12]. The ACGME website was used to access program websites via the link provided. If no link was provided on the ACGME page or the link was nonfunctional, the program website was reached via Google search with “program name + colorectal fellowship” as the search parameters. Websites were accessed from a US internet service provider between October 3 and November 27, 2019.

### Outcome Measured

Each program website was evaluated by two independent reviewers who were blinded to the other’s scores. A validated website assessment tool was used, including an interactive index and an information index [7]. The interactive index encompassed accessibility, design, organization, and user-friendliness. Accessibility was graded out of 3 points: 1 point for having a link on the ACGME website, 1 point if the link provided was functional, and 1 point if the link led directly to the colorectal fellowship webpage. Design, organization, and user-friendliness were graded on a 4-point scale: 1, poor; 2, acceptable; 3, good; and 4, excellent. An information index was created to quantify 64 content items known to be valued by applicants. Content was evaluated in the categories of application (13 items), education (24 items), personnel (13 items), and benefits (14 items; see Multimedia Appendix 1 for definitions of each content item). Each content item was weighted equally. If a content item was found on the main webpage of the fellowship program or through a direct link on the main webpage, it was awarded 1 point. If the information was incomplete (eg, different types of conferences were listed but frequency—day of the week, every week or once a month, etc—was not included) or found through a separate website (eg, benefits and salaries listed on the GME site or faculty profiles listed on the departmental site), it was awarded 0.5 points. When reporting the percentage of websites containing a specific information, websites that scored 0.5 or 1 were both considered as having the information. If the information was unavailable, it was awarded 0 points. Overall, websites could receive 15 points for the interactive index and 64 points for the information index, for a maximum total score of 79 points.

### Rater Training and Performance

Reviewers were given detailed definitions of each scoring criteria and trained using a sample of 10 general surgery residency websites of various quality. Interrater correlation coefficient for total score was 0.94 (95% CI 0.91-0.97). Rater agreement was 81% and weighted Kappa was 0.74. If there was disagreement between two reviewers regarding accessibility or content items in the information index, it was reviewed, and consensus was reached. For design, organization, and user-friendliness, the average score of two raters was used as the final score.

### Analysis of the Outcomes

Continuous variables were reported as median (IQR) values. Categorical variables were reported as count (percentage) values. An analysis of programs based on four main geographic locations, as defined by the United States Census Bureau (Northeast, South, Midwest, and West), was performed using a Kruskal-Wallis test for continuous variables and chi-square for categorical variables. Association between two continuous variables was assessed by Kendall correlation coefficient. Statistical analysis was performed in RStudio version 1.2.5001 (RStudio, Inc).

### Institutional Review Board and Ethics Statement

All data reviewed was open to the public, and there was no contact with fellowship staff; thus, no institutional review board review, ethics approval, or informed consent was necessary.

## Results

### Overall Performance

There was a total of 63 ACGME-accredited colorectal fellowship programs in the United States. Of 63 programs, 5 (9%) did not have a functional website (in spite of being established prior to 2012) and thus were excluded from analysis. Of the 58 programs with a functional website, the median total score was 27.8 (IQR 21.5-34.5) of 79. When stratified by geographic location, there were 21 programs in the Northeast, 18 in the Midwest, 17 in the South, and 7 in the West. There was no significant difference in the total score of programs in different geographic regions within the United States (*P*=.46). There was no correlation between age of the program and total score (*P*=.38). No program had a Facebook profile or Instagram account to promote their fellowship.

### Interactive Index

Programs scored a median of 7.5 (IQR 6.0-10.0) of 15.0 for the interactive index, including design (median 2.0, IQR 1.5-2.5 of 4), organization (median 2.0, IQR 1.0-2.5 of 4), user-friendliness (median 2.0, IQR 1.0-2.5 of 4) and accessibility (median 2.0, IQR 2.0-2.0 of 3.0). For accessibility, the ACGME website provided a website link for 53 of 58 (91%) programs. Of those 53 programs with links, only 85% (n=45) of those links were functional, and only 31% (n=14) of the functional links led directly to the colorectal fellowship webpage; the remaining functional links led to a general departmental page.

### Information Index

Programs scored a median of 20.0 (IQR 14.1-24.5) of 64 possible points for the information index content items. When further broken down, the median score (IQR) was 5.0 (4.0-6.5) of 13.0 for application information, 5.5 (3.1-9.0) of 24.0 for education information, 3.3 (2.5-5.0) of 13.0 for personnel information, and 4.0 (1.6-6.8) of 14.0 for benefits information. Over one-third (20/58) of programs received less than 16 points, while only 2 (3.4%) programs received 32 points or more for the presence of the information index content items.

**Figure 1:**
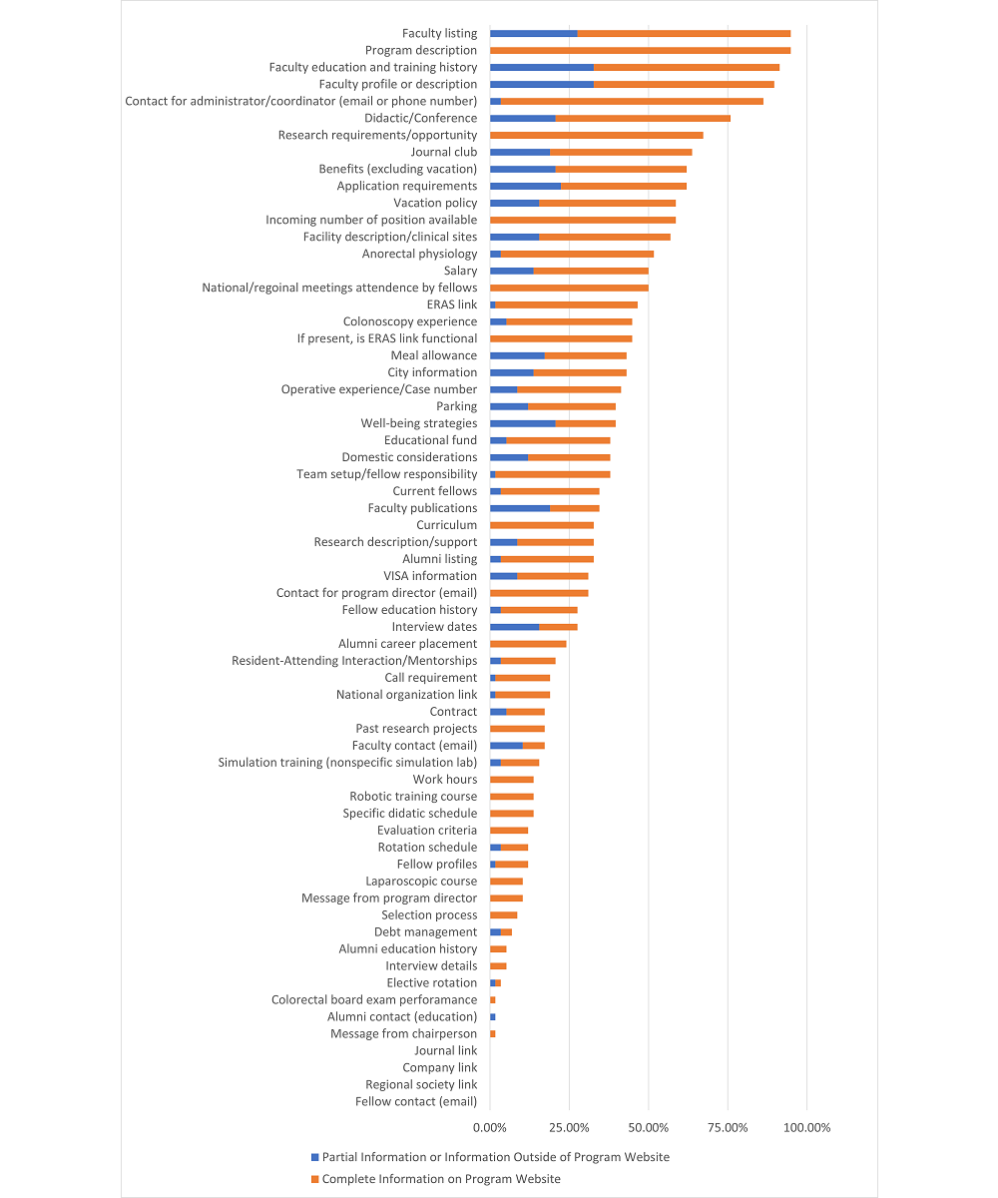
Program website content items across 58 colorectal programs. ERAS: Electronic Residency Application Service.

Only 59% (34/58) of the functional fellowship websites disclosed the number of fellowship positions available (Figure 1). Although 86% (50/58) of programs provided contact information for program administrative staff, only 31% (18/58) provided contact information for the program director. Most programs (50/58, 86%) identified their program director. A total 40% (23/58) of programs presented detailed fellowship application requirements and a list of documents required for a complete application, while 22% (13/58) offered only general eligibility criteria. Few programs provided detailed information on applicant selection criteria or interview information.

Most of the 58 colorectal fellowship websites provided faculty listings (n=55, 95%), information on the education of their faculty (n=53, 91%), and dedicated faculty profiles (n=52, 90%). However, 28% (n=16) of faculty listings and 33% (n=19) of faculty profiles were on the general departmental website without a direct link from the specific fellowship website. Only 34% (n=20) of websites listed the current fellows and 33% (n=19) listed information on their alumni. Contact information for faculty (n=10, 17%), fellows (n=0, 0%), and alumni (n=1, 2%) was rarely reported publicly on the websites.

Most (n=44, 76%) of the 58 programs provided a list of conferences and didactic education, with 55% (n=32) of programs reporting frequency of these conferences. Journal club was mentioned by 64% (n=37) of programs. Although research requirements or opportunities were listed for 67% (n=39) of the programs, a description of the research or potential research support resources were only available for 33% (n=19) of the programs; past research projects were listed on 17% (n=10) of the sites. Only 33% (n=19) of programs provided operative caseload of the fellows, while an additional 9% (n=5) listed examples of operations without case volume. Colonoscopy volume or time allocated to endoscopy was provided by 45% (n=26) of the program websites and anorectal physiology was mentioned by 52% (n=30) of the programs. Half of the programs had information regarding expectations on national or regional meeting attendance by fellows. Only 1 (2%) program provided any information regarding the colorectal board performance of prior fellows.

Most (n=48, 83%) of the 58 websites provided some information regarding employment benefits or practice location lifestyle. Benefits (n=36, 62%), vacation policy (n=34, 59%), and salary (n=29, 50%) were the three most common benefit or lifestyle content items provided by programs. Debt management (n=4, 7%), work hours (n=8, 14%), and a sample contract (n=10, 17%) were the three least common benefit or lifestyle content items reported by programs. About one-third of the websites that provided benefit or lifestyle information had to be found on the associated GME page via search.

## Discussion

### Principal Results

Colorectal fellowship programs have lagged in embracing program websites as a recruitment tool despite the ubiquitous use of program websites throughout the application process [3,4]. A total 9% (5/63) of programs did not have a functional program website. Programs that had a functional website frequently were difficult to access, not user-friendly, and lacked applicant valued content.

### Limitations

This study has several limitations. This is a cross-sectional study. It is possible that program websites were altered after data collection. However, data was collected from September to November 2019. This period of time encompasses the fellowship application season and should reflect what applicants were able to access in the 2019 application cycle. Additionally, no survey of colorectal fellowship applicants was performed to identify what that particular pool of applicants finds important on program websites. This information was inferred from the literature where studies have been performed for other specialties. However, each applicant likely has their own perception on what information is considered valuable. Thus, it would benefit both the programs and applicants for these websites to be as comprehensive as possible. Information was evaluated as being either present or absent, but it could not be verified if the information was current and accurate. Lastly, we were not able to evaluate changes in program websites over a period of time, as this was a cross-sectional study performed in 2019.

### Comparison With Prior Work

In this study, it was unexpectedly found that 5 of 63 (9%) colorectal fellowship programs did not have an accessible website. In the current era, all programs should have a functional website. Studies from other specialties have revealed an absence of program websites for less than 1% of radiology programs to as high as 30% of pediatric orthopedic surgery programs [3,8-11,13,14]. Fellowship programs, as compared to residency programs, tend to have a greater percentage of websites that are not regularly maintained. Silvestre et al [8] postulated that a subspecialty within a specialized field may be a smaller community where word of mouth and reputation play a bigger role than online presence. Studies as early as 1999, however, have shown that 1 out of 7 applicants would rank programs without a website lower than those with a website [1]. Programs without websites risk losing highly competent applicants. Additionally, Instagram and Facebook are great avenues for both education [15] and fellowship program promotion; however, this is not used by any colorectal fellowship program.

Program websites should not merely exist but should be easily accessible and user-friendly. One-fifth of the links provided on the ACGME webpage were not functional, and only a quarter led directly to the fellowship website. Programs should check and update links on the ACGME website and other major program listing sites on an annual basis. Additionally, applicants value ease of navigation more than the presence of individual content items [4]. In this study, many websites were disorganized and lacked links to allow easy navigation between content domains such as the GME webpage and colorectal department webpage. Program websites ought to provide direct links to the desired content and design an interface that is easy to interact with, allowing users to find information quickly [6].

Only a quarter of the content items we evaluated were presented by half or more programs. Information regarding application process; current faculty, residents, or alumni; and training information are highly valued by applicants [2,3,5]. Interview dates and details are incredibly important for fellowship applicants, as senior residents must balance time spent interviewing with a heavy clinical workload, but was only provided by 5% (29/58) of the programs. Surprisingly, 14% (8/58) of colorectal programs, double that reported by plastic surgery, did not list contact information for administrative staff, denying the possibility of reaching out when applicants have questions [9]. Personal profiles of current faculty and fellows can help applicants gauge their self-perceived fit with a program, influencing where they choose to apply and interview. However, this information is frequently missing on colorectal program websites. Even though information regarding education and training is the most important content for applicants, the program with the most information about education and training reported less than half of the content items evaluated in our study. Lastly, quality of life is an important driver in career choice and may be viewed as a *sensitive topic* not discussed during an interview [7,10,16]. Thus, it is important for program websites to provide such information.

Although this analysis is limited to colorectal fellowships, it serves as an alarm that current training program websites are not meeting the needs of applicants. Although the program websites of some specialties have been reviewed and showed similar deficiency in quality, there exists a gap in many specialties where program websites have not been reviewed. Thus, all training programs, regardless of specialties, should maintain a program website that is updated before each application cycle. Direct links to the program website should be updated on all major listings such as the ACGME program list, FRIEDA, or the Electronic Residency Application Service. Information available on the departmental webpage or GME webpage should be connected to the fellowship program website via a direct link. Programs should optimize the design, organization, and usability of their websites by grouping information (when appropriate), using hyperlinks to lead applicants to the departmental webpage or GME webpage, and separating content in a way that maintains easy navigation between pages. Future studies that survey applicants of different specialties or subspecialties on their specific needs will further clarify the important content to include that may be unique to each specialty. Guidelines from GME or professional societies would also provide guidance for individual programs in the development of a high-quality program website.

### Conclusions

This study found the overall quality of colorectal fellowship program websites to be poor. Colorectal fellowship programs need to increase the amount of information available and improve the usability of their websites. Absent or poorly designed fellowship websites can negatively impact the application and recruitment process for both applicants and fellowship programs, as both seek to find the best fit for the program.

## Appendix

Multimedia Appendix 1 Definition and scoring criteria for information index.

## Abbreviations

ACGME: Accreditation Council for Graduate Medical Education
GME: graduate medical education
