# Supplementary material for: Critical Evaluation of the Efficiency of Colorectal Fellowship Websites: Cross-sectional Study
Source: JMIR Med Educ. 2021 Oct 15;7(4):e30736. doi: 10.2196/30736 (PMC8556633; doi:10.2196/30736)
Supplement: Multimedia Appendix 1 [file mededu_v7i4e30736_app1.pdf]

Question	Score		
**Application**			
Number of incoming positions provided?	1-Yes	0.5-Partial	0-No
Contact information for PD (email) provided?	1-Yes	0.5-Partial	0-No
Contact information for administrator/coordinator (email or office number) provided?	1-Yes	0.5-Partial	0-No
Program description available?	1-Yes	0.5-Partial	0-No
Facility description/ clinical sites available?	1-Yes	0.5-Partial†	0-No
Message from the program director available?	1-Yes	0.5-Partial	0-No
Message from the chairperson available?	1-Yes	0.5-Partial	0-No
Application requirement available?	1-Yes	0.5-Partial†	0-No
Selection process described?	1-Yes	0.5-Partial	0-No
Interview dates provided?	1-Yes	0.5-Partial†	0-No
Interview details provided?	1-Yes	0.5-Partial	0-No
ERAS link provided?	1-Yes	0.5-Partial	0-No
If present, is ERAS link functional?	1-Yes	0.5-Partial	0-No
**Personnel**			
Faculty listing provided?	1-Yes	0.5-Partial	0-No
Faculty education and training history provided?	1-Yes	0.5-Partial	0-No
Faculty profile or description provided?	1-Yes	0.5-Partial	0-No
Faculty publications provided?	1-Yes	0.5-Partial	0-No
Faculty contact info (email) provided?	1-Yes	0.5-Partial	0-No
Current fellow listing provided?	1-Yes	0.5-Partial	0-No
Fellow education history provided?	1-Yes	0.5-Partial	0-No

Fellow profiles or descriptions provided?	1-Yes	0.5-Partial	0-No
Fellow contact information (email) provided?	1-Yes	0.5-Partial	0-No
Alumni listing provided?	1-Yes	0.5-Partial	0-No
Alumni education history provided?	1-Yes	0.5-Partial	0-No
Alumni contact information (email) provided?	1-Yes	0.5-Partial	0-No
Alumni career placement provided?	1-Yes	0.5-Partial	0-No
**Education**			
Colorectal board exam performance provided?	1-Yes	0.5-Partial	0-No
Program setup described (e.g. set up of services, components of careteam, team members responsibility, etc)?	1-Yes	0.5-Partial	0-No
Curriculum described?	1-Yes	0.5-Partial	0-No
Didactic/Conference described?	1-Yes	0.5-Partial†	0-No
Specific didactic schedule present?	1-Yes	0.5-Partial	0-No
Laparoscopic course or training offered?	1-Yes	0.5-Partial	0-No
Robotic training course or certificate offered?	1-Yes	0.5-Partial	0-No
Non-specified simulation training offered?	1-Yes	0.5-Partial	0-No
Colonoscopy experience described?	1-Yes	0.5-Partial†	0-No
Ano-rectal physiology experience described?	1-Yes	0.5-Partial†	0-No
Operative experience (case volume) provided?	1-Yes	0.5-Partial†	0-No
Rotation Schedule provided?	1-Yes	0.5-Partial	0-No
Availability of elective rotation provided?	1-Yes	0.5-Partial	0-No
Research requirements or opportunities described?	1-Yes	0.5-Partial	0-No

Research support or area of focus described?	1-Yes	0.5-Partial	0-No
Past research projects described?	1-Yes	0.5-Partial	0-No
Journal club availability and frequency?	1-Yes	0.5-Partial†	0-No
National/regional meetings attendance by fellows provided?	1-Yes	0.5-Partial	0-No
Evaluation criteria provided?	1-Yes	0.5-Partial	0-No
National organization links available?	1-Yes	0.5-Partial	0-No
Regional society links available?	1-Yes	0.5-Partial	0-No
Company links available?	1-Yes	0.5-Partial	0-No
Journal links available?	1-Yes	0.5-Partial	0-No
Resident-attending interactions or mentorships described?	1-Yes	0.5-Partial	0-No
**Benefits and lifestyle**			
Call requirement provided?	1-Yes	0.5-Partial†	0-No
Contract sample provided?	1-Yes	0.5-Partial	0-No
Work hours limit provided?	1-Yes	0.5-Partial	0-No
Benefits (medical insurance, retirement account, etc) provided?	1-Yes	0.5-Partial†	0-No
Vacation policy provided?	1-Yes	0.5-Partial	0-No
City information provided?	1-Yes	0.5-Partial	0-No
Domestic considerations (childcare, residence consideration, etc) described?	1-Yes	0.5-Partial	0-No
Wellbeing strategies provided?	1-Yes	0.5-Partial	0-No
Salary information provided?	1-Yes	0.5-Partial	0-No
Debt management/resident finances provided?	1-Yes	0.5-Partial	0-No
Meal allowance or on-call meal provided?	1-Yes	0.5-Partial	0-No

Educational funds or resources described?	1-Yes	0.5-Partial	0-No
Parking provided?	1-Yes	0.5-Partial	0-No
VISA information or citizenship requirement described?	1-Yes	0.5-Partial	0-No

e-Appendix. Definition and scoring criteria for information index.

Partial point - 0.5 was given for information found on the institution website outside of the colorectal fellowship page.

† 0.5 points given also for information present that lack detail including: listed facilities but no description; gave general eligibility but no listing of specific requirements; interview months provided but not specific dates; didactics or conference schedule listed but frequency not provided; subjective description of colonoscopy experience without specific time allocation or volume; mentioned ano-physiology lab without specific detail of the range of tests performed or specific time allocation; provided hospital caseload or listed surgeries performed but not caseload of an individual graduate; provided information on the presence of Journal Club without frequency; specified whether call was required but no information regarding the frequency or nature of call; benefits listed but no link to any additional detail.
